# Azacitidine-induced reconstitution of the bone marrow T cell repertoire is associated with superior survival in AML patients

**DOI:** 10.1038/s41408-022-00615-7

**Published:** 2022-01-28

**Authors:** Juliane Grimm, Donjete Simnica, Nadja Jäkel, Lisa Paschold, Edith Willscher, Susann Schulze, Christine Dierks, Haifa Kathrin Al-Ali, Mascha Binder

**Affiliations:** 1 Department of Internal Medicine IV, Oncology/Hematology, University Hospital Halle (Saale), Halle (Saale), Germany; 2 Department of Internal Medicine IV, Oncology/Hematology, Martin-Luther-University Halle-Wittenberg, Halle (Saale), Germany; 3 Krukenberg Cancer Center, University Hospital Halle (Saale), Halle (Saale), Germany

**Keywords:** Immunosurveillance, T-cell receptor, Acute myeloid leukaemia

## Abstract

Hypomethylating agents (HMA) like azacitidine are licensed for the treatment of acute myeloid leukemia (AML) patients ineligible for allogeneic hematopoietic stem cell transplantation. Biomarker-driven identification of HMA-responsive patients may facilitate the choice of treatment, especially in the challenging subgroup above 60 years of age. Since HMA possesses immunomodulatory functions that constitute part of their anti-tumor effect, we set out to analyze the bone marrow (BM) immune environment by next-generation sequencing of *T cell receptor beta* (*TRB*) repertoires in 51 AML patients treated within the RAS-AZIC trial. Patients with elevated pretreatment T cell diversity (11 out of 41 patients) and those with a boost of *TRB* richness on day 15 after azacitidine treatment (12 out of 46 patients) had longer event-free and overall survival. Both pretreatment and dynamic BM T cell metrics proved to be better predictors of outcome than other established risk factors. The favorable broadening of the BM T cell space appeared to be driven by antigen since these patients showed significant skewing of *TRBV* gene usage. Our data suggest that one course of AZA can cause reconstitution to a more physiological T cell BM niche and that the T cell space plays an underestimated prognostic role in AML.

**Trial registration**: DRKS identifier: DRKS00004519

## Introduction

Acute myeloid leukemia (AML) remains a fatal disease with only around 10–15% long-term survival in patients older than 60 years [1, 2]. For eligible patients, the standard treatment is based on intensive chemotherapy and has hardly changed throughout the last decades. The established concept of allogeneic hematopoietic stem cell transplantation (HSCT) already proved AML to be curable with a T cell-based immunotherapy [3, 4]. The fact that the graft-versus-leukemia (GvL) effect is able to permanently eradicate residual AML cells may also point towards impaired immunosurveillance with defective T cell responses to be a crucial part of leukemogenesis.

In general, AML is characterized by an immunosuppressive microenvironment with multiple mechanisms being involved in immune escape of AML blasts: Part of it is thought to be mediated by soluble factors such as cytokines since some signatures have been shown to be associated with overall survival in AML patients [5–8]. Other mechanisms of impaired immunosurveillance in AML rely on circumventing target recognition and subsequent cytotoxic T cell responses, *e.g*. by loss of HLA class II expression on the AML blasts after allogeneic HSCT [9]. T cell suppression can also be mediated by increased expression of surface markers inhibiting immune responses (*e.g*. LILRB4 or PD-L1) [10, 11]. As a consequence, AML patients—and especially patients with refractory disease or relapse after HSCT—display higher frequencies of exhausted T cells compared to healthy donors [12–19]. The malfunctioning T cell space in AML is also characterized by a general down-regulation of genes involved in T cell activation [20], as well as a skewed T cell repertoire with reduced diversity compared to healthy donors [19, 21, 22].

In light of the dysfunctional T cell space in AML, therapeutic approaches reconstituting immunosurveillance apart from allogeneic HSCT—which remains a treatment option essentially restricted to patients with limited comorbidities—are desirable. There is emerging evidence that hypomethylating agents (HMA) such as azacitidine (AZA) possess immunomodulatory functions that may constitute part of their anti-tumor effect. These range from upregulation of epigenetically silenced tumor antigens [23, 24] and interferon signaling [25] to promote T cell proliferation [26, 27] while decreasing the number of T_regs_ [25, 28]. But also immunosuppressive functions have been described, such as PD-L1 upregulation and inhibition of T cell activation [29, 30]. HMA are currently licensed as first-line therapy for AML patients ineligible for HSCT because of disease- and/or patient-related factors [31]. Yet, reliable biomarkers facilitating the choice of treatment especially in elderly patients above 60 years of age and borderline performance status are largely lacking. Further insight into the immunomodulatory mechanisms of these agents may help to identify immunological signatures predicting response to standard induction vs hypomethylating treatment that could help to guide treatment decisions in this challenging patient population.

We predicted that certain characteristics of the bone marrow (BM) T cell space may provide a “fertile ground” for the immunomodulatory mechanism of action of HMA. To test this, we studied pretreatment and matched early-on-treatment BM immune signatures derived from next-generation sequencing (NGS) of the *T cell receptor beta* (*TRB*) repertoire in an AML cohort homogenously starting their systemic treatment with AZA within the RAS-AZIC study of the East German Study Group for Hematology and Oncology (OSHO)—an investigator-initiated multicenter trial combining AZA and standard chemotherapy in a sequential response-adapted design (OSHO #83; DRKS00004519) [32].

## Materials and methods

### Patient characteristics

The study was performed as part of the investigator-initiated multicenter RAS-AZIC trial (DRKS00004519) which combined AZA treatment with standard intensive chemotherapy in a response-based sequential approach in elderly AML patients (age ≥60 years). The study design is displayed in Supplemental Fig. 1.

In this translational project, we analyzed BM aspirates of a sub-cohort of the RAS-AZIC trial comprising 51 AML patients. The here analyzed patients were enrolled in the study between July 2013 and April 2016. Pretreatment samples of 41 patients and early-on-treatment BM samples (day 15) of 46 patients were available with 36 patients having eligible material at both time points. All patients had previously untreated *de novo* (*n* = 28, 54.9%) or secondary AML (*n* = 23, 45.1%). AML was diagnosed based on the presence of ≥20% myeloid blasts in the BM in one of the following diagnostic approaches: morphologic assessment of BM smears, histologic samples, or flow cytometry-based immunophenotyping. The blast counts displayed throughout the manuscript result from morphologic assessment of BM smears.

The patients were homogeneously treated with one course of AZA (75 mg/m^2^/day s.c. for 7 days) and subsequent treatment depended on the BM blast count on day 15. Patients with blasts <45% continued with AZA treatment on day 28 while those with blasts ≥45% received intensive chemotherapy (Mitoxantrone 10 mg/m^2^/day d1–3 and cytarabine 1 g/m^2^/BID d1, 3, 5, 7) on day 17. Supplemental Table 1 summarizes the clinical characteristics of the patients analyzed in this sub-study. As a control, BM of healthy donors (HD) without any hematological abnormalities was obtained from our AML biobank. All patients and HD analyzed in this study gave their written informed consent for the use of their biological material for scientific research. The study was approved by the ethics committee and was performed in concordance with the Declaration of Helsinki.

### Isolation of genomic DNA

Mononuclear cells were isolated from BM by density-gradient centrifugation using Ficoll^®^ solution. Genomic DNA was then isolated using the automated QIAcube platform (Qiagen, Hilden, Germany) in accordance with the manufacturer’s instructions.

### Amplification and NGS of the TRB repertoire

The rearranged *V*, *D*, and *J* gene segments of the *TRB* locus were amplified together in a multiplex PCR using *TRB-A/-B* primer pools and 250 ng of genomic DNA [33]. The primers were purchased from Metabion International AG (Martinsried, Germany). As described in Schliffke et al. [34], two consecutive PCR reactions were performed to generate *TRB* fragments tagged with Illumina-compatible adapters for hybridization to the flow cell and seven nucleotide barcodes for sample identification. All PCRs were performed using Phusion HS II (Thermo Fisher Scientific Inc., Darmstadt, Germany). After gel electrophoretic separation, *TRB* amplicons were purified using the NucleoSpin^®^ Gel and PCR Clean-up kit (Macherey-Nagel, Düren, Germany), quantified on the Qubit platform (QIAGEN, Hilden, Germany), and pooled to a final concentration of 8 nM. The quality of the *TRB* amplicon pools was controlled on an Agilent 2100 Bioanalyzer (Agilent Technologies, Böblingen, Germany) before undergoing NGS. The samples were sequenced with a mean sequencing depth of 80,520 reads (range 42,440–137,852 reads).

NGS and demultiplexing was performed on an Illumina MiSeq sequencer (600-cycle single indexed, paired-end run, V3-chemistry). Analysis of the rearranged *TRB* loci was computed and plotted as previously described [35, 36].

### Immune repertoire metrics

Clonality, richness, and diversity as basic immune repertoire metrics were determined as previously described [35, 36]. Clonal space analyses were carried out using the packages tcr [37], and tidyverse [38], and bubble plots were created using the packages packcircles and ggplot2 in R.

### In silico GLIPH2 and generation probability analysis

We used the GLIPH2 (grouping of lymphocyte interactions by paratope hotspots) algorithm to cluster *TRB* sequences that share antigen specificity with a high likelihood [39]. Clusters are displayed as consensus sequences derived from the unique complementarity-determining region 3 (CDR3) amino acid sequences the respective cluster is composed of. The mean CDR3 amino acid frequency is considered as “cluster size”. The generation probability of the T cell clusters was calculated using the OLGA (optimized likelihood estimate of immunoglobulin amino acid sequences) algorithm [40]. All values are log_2_ transformed for plotting purposes.

### Statistical analyses

Differences in NGS metrics were studied by student’s *t*-test. Principal component analysis (PCA) differences were identified by the Pillai–Bartlett test of MANOVA. Survival analyses were calculated using the Kaplan–Meier method and groups were compared with the log-rank test. For further details on clinical endpoint definition and multivariate analyses see Supplemental Data. All statistical analyses were performed using GraphPad Prism 9.0.1 (GraphPad Software, La Jolla, CA, USA) and the R statistical software platform (version 3.6.3).

## Results

### Deep sequencing of TRB repertoires in AML patients treated with AZA

**Fig. 1:**
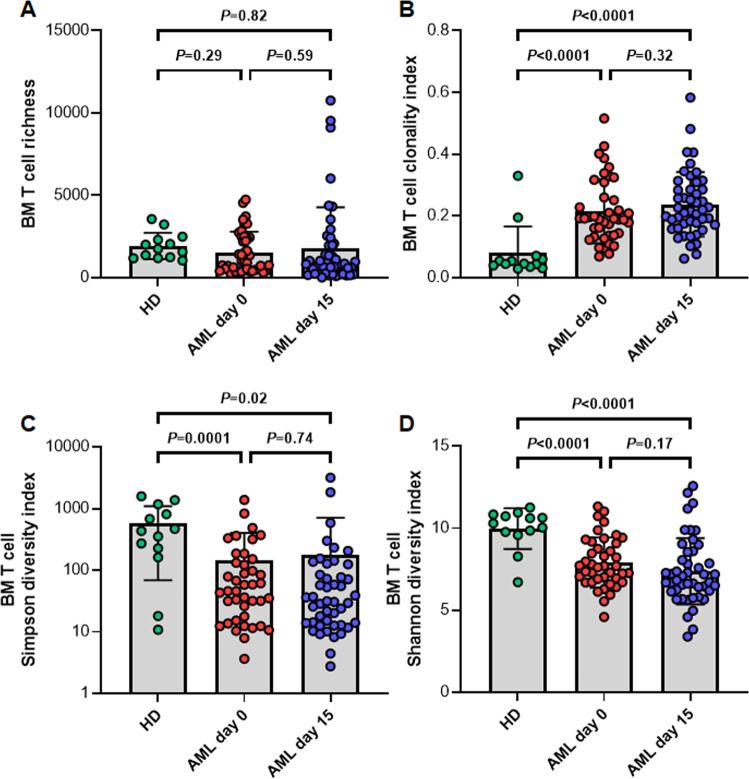
Global immune metrics of the entire cohort. The figure displays the bone marrow (BM) T cell receptor repertoire richness (**A**), clonality (**B**), Simpson (**C**), and Shannon indices (**D**) as measures for the repertoire diversity for the healthy control group, as well as the AML samples before treatment initiation (AML day 0) and after the first course of azacitidine (AML day 15).

To gain insights into the T cell BM niche of AML patients and its potential transformation through treatment with an HMA, we performed immuno-NGS on BM T cells of 51 AML patients as well as 13 HD. The latter served as donors in the context of an allogeneic HSCT and this cohort was matched to the sex and age distribution in the AML cohort. While T cell richness did not significantly differ between AML and HD BM *TRB* repertoires (d0 – *P* = 0.29; d15 – *P* = 0.82; Fig. 1A), AML patients displayed a more clonal (d0 – *P* < 0.0001; d15 – *P* < 0.0001; Fig. 1B) and less diverse T cell space (Simpson diversity Index: d0 – *P* = 0.0001; d15 – *P* = 0.02; Shannon diversity Index: d0 – *P* < 0.0001; d15 – *P* < 0.0001; Fig. 1C, D). However, we did not observe significant differences in global immune metrics between AML patients before and after AZA treatment (Fig. 1). Additionally, we analyzed the length of the CDR3 sequence as an additional marker for the diversity of the *TRB* repertoire. CDR3 length displayed Gaussian distribution without relevant differences between AML patients pre- and post-AZA (Supplemental Fig. 2).

### Association of pretreatment BM TRB repertoire diversity with survival

**Fig. 2:**
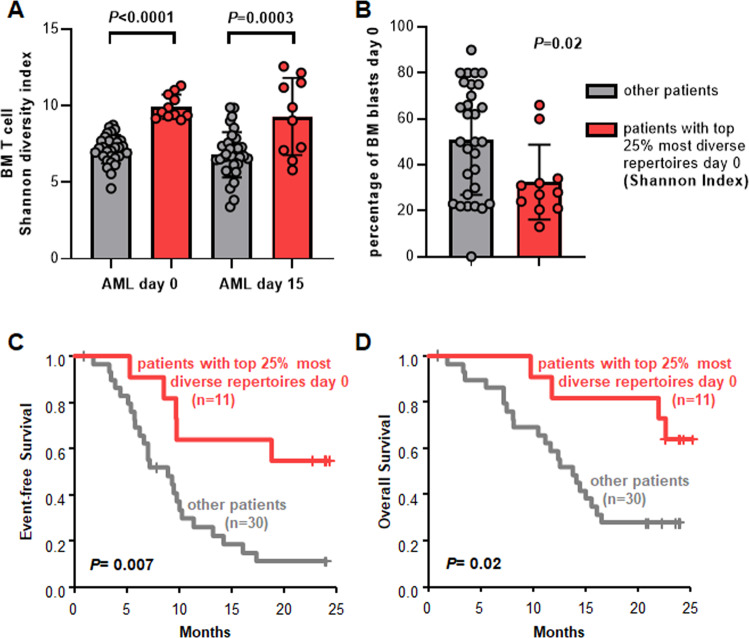
AML patients with the most diverse bone marrow (BM) T cell receptor beta (TRB) repertoires at diagnosis harbor favorable prognosis compared to the other patients. Applying the third quartile of the measured Shannon indices as cut-off we identified a group of AML patients with particularly diverse *TRB* repertoires (**A**), who had fewer bone marrow blasts (**B**) and displayed prolonged event-free (**C**), and overall survival (**D**).

We observed that some AML pretreatment samples displayed diversity indices similar to the metrics observed in BM of healthy individuals. We, therefore, used the third quartile of the measured Shannon diversity indices to differentiate AML patients with highly diverse from patients with less diverse repertoires. When applying this cut-off, the median Shannon diversity index of the identified highly diverse AML *TRB* repertoires was comparable to that of HD (9.59 vs 10.29; *P* = 0.94). Clinical characteristics of AML patients with high or low diversity of the T cell space were largely similar except for higher BM blast counts in the latter (Table 1 and Fig. 2B, *P* = 0.02). In the analyzed AML cohort treated with HMA, a highly diverse pretreatment T cell space – as estimated with Shannon diversity index—was associated with longer event-free (*P* = 0.007; Fig. 2C) and longer overall survival (*P* = 0.02; Fig. 2D). We obtained similar results when we excluded patients who died within the first 90 days of the trial (Supplemental Fig. 3) or when we applied the Simpson index which is a diversity index adding less weight to smaller T cell clones confirming the robustness of our data (Supplemental Fig. 4 and Supplemental Table 2). On the contrary, a high pretreatment BM blast count alone did not significantly associate with survival measures in the analyzed AML cohort (Supplemental Fig. 5). Similarly, other established risk factors, e.g., age, disease origin (de novo vs secondary AML), and genetic markers did not significantly associate with outcome in this cohort treated within the trial (Supplemental Fig. 6) and the proportion of patients receiving allogeneic HSCT was comparable between patients with more and less diverse *TRB* repertoires (Table 1). This suggests that pretreatment T cell microenvironment metrics might be a better predictor of survival endpoints than conventional risk factors in this specific subset of AML patients under these treatment conditions.

**Table 1:** Clinical characteristics of AML patients treated with azacitidine according to the diversity of their pretreatment BM T cell repertoire based on the Shannon diversity index.

	AML patients with top 25% most diverse repertoires at day 0 (*n* = 11)	other AML patients (*n* = 30)	*P*^a^
age at diagnosis median (range)	68.4 (60.8–80.9)	69.4 (61.0–83.6)	0.72
AML origin, *n* (%)			
de novo	6 (54.5)	18 (60.0)	1
secondary	5 (45.5)	12 (40.0)	
BM blasts at day 0, % median (range)	28 (13–66)	50 (0–90)	0.02
BM blasts at day 15, % median (range)	15 (6–72)	30 (9–72)	0.03
*NPM1* mutation			
present	1 (9.1)	8 (26.7)	0.40
absent	10 (90.9)	22 (73.3)	
*FLT3*-ITD mutation			
present	0 (0.0)	7 (23.3)	0.16
absent	11 (100.0)	23 (76.7)	
normal karyotype			
present	5 (45.5)	14 (46.7)	0.39
absent	5 (45.5)	16 (53.3)	
unknown	1 (9.1)	0 (0.0)	
complex karyotype			
present	2 (18.2)	4 (13.3)	0.20
absent	8 (72.7)	26 (86.7)	
unknown	1 (9.1)	0 (0.0)	
allogeneic HSCT			
performed	5 (45.5)	12 (40.0)	1
not performed	6 (54.5)	18 (60.0)	

*AML* acute myeloid leukemia, *BM* bone marrow, *NPM1* *nucleophosmin 1*, *FLT3* *fms related receptor tyrosine kinase 3*, *ITD* internal tandem duplication, *HSCT* hematopoietic stem cell transplantation.

^a^*P* values are from Fisher’s exact or Kruskal–Wallis test and compare the two groups.

### Early broadening of the T cell space after AZA treatment is associated with a favorable prognosis

**Fig. 3:**
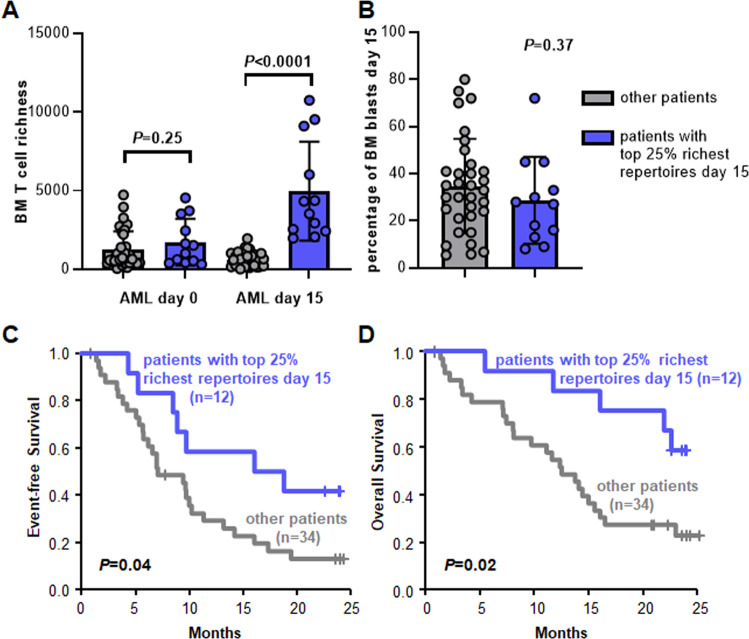
AML patients who experience a richness boost of their bone marrow (BM) T cell receptor beta (TRB) repertoires after the first course of azacitidine have superior prognosis. Applying the third quartile of the measured richness as cut-off we identified AML patients with extremely rich *TRB* repertoires after azacitidine treatment (**A**), without any association to the measured blast count (**B**), and longer event-free (**C**), as well as overall survival (**D**) compared to the other patients.

Although BM *TRB* repertoire richness did not significantly differ between AML patients and HD, some AML patients experienced a boost in T cell richness after the first course of AZA. We, therefore, applied the third quartile of the determined richness values on day 15 after the first AZA cycle to define AML patients responding to AZA treatment by broadening of their T cell space (Fig. 3A). We could not identify any clinical characteristics to be predictive of this immunological response to AZA (Table 2). Of note, this response pattern was not associated with early blast clearance (Fig. 3B). The rise in T cell richness on day 15 was accompanied by significantly increased diversity indices compared to the other patients (median Shannon diversity index day 15: 6.64 vs 9.88; *P* < 0.0001; Supplemental Fig. 7). When we analyzed the pretreatment global immune metrics of the patients with particularly high *TRB* repertoire richness on day 15, we observed that these patients already showed increased *TRB* diversity and decreased clonality before the first course of AZA compared to the rest of the cohort (median Shannon diversity index day 0: 8.64 vs 7.28, *P* = 0.05; clonality index day 0: 0.14 vs 0.21, *P* = 0.05; Supplemental Fig. 7). AML patients who experienced a richness boost of their BM T cell space after the first course of AZA had significantly longer event-free (*P* = 0.04; Fig. 3C) and overall survival (*P* = 0.02; Fig. 3D) compared to the patients who did not show this immunological response. When excluding AML patients who died early (within the first 90 days of the trial) we still observed prolonged survival for the patients experiencing a broadening of their BM T cell space (Supplemental Fig. 8). To investigate the impact of an allogeneic HSCT and other clinical features on the prognostic value of the immunological response after AZA, we performed multivariate analysis. After forward adjusting the application of an allogeneic HSCT was not included in the final models for event-free and overall survival based on the Bayesian information criterion and the BM T cell richness on day 15 remained the only significant prognostic factor for event-free survival and was positively impacted by trend on overall survival after adjusting for the presence of a normal karyotype and the platelet count at diagnosis (Supplemental Table 3).

**Table 2:** Clinical characteristics of AML patients according to the richness of their pretreatment bone marrow T cell repertoire after the first course of azacitidine (day 15).

	AML patients with top 25% richest repertoires (*n* = 12)	other AML patients (*n* = 34)	*P*^a^
age at diagnosis median (range)	65.6 (60.8–80.9)	70.5 (60.9–83.5)	0.23
AML origin, *n* (%)			
de novo	8 (66.7)	17 (50.0)	0.50
secondary	4 (33.3)	17 (50.0)	
BM blasts at day 0, % median (range)	33 (0–80)	47.5 (0–90)	0.48
BM blasts at day 15, % median (range)	27.5 (8–72)	33 (5.4–80)	0.37
*NPM1* mutation			
present	3 (25.0)	7 (20.6)	0.70
absent	9 (75.0)	27 (79.4)	
*FLT3*-ITD mutation			
present	3 (25.0)	4 (11.8)	0.36
absent	9 (75.0)	30 (88.2)	
normal karyotype			
present	5 (41.7)	16 (47.1)	0.39
absent	6 (50.0)	18 (52.9)	
unknown	1 (8.3)	0 (0.0)	
complex karyotype			
present	3 (25.0)	3 (8.8)	0.06
absent	8 (66.7)	31 (91.2)	
unknown	1 (8.3)	0 (0.0)	
allogeneic HSCT			
performed	7 (58.3)	10 (29.4)	0.09
not performed	5 (41.7)	24 (70.6)	

*AML* acute myeloid leukemia, *BM* bone marrow, *NPM1* *nucleophosmin 1,* *FLT3* *fms related receptor tyrosine kinase 3*, *ITD* internal tandem duplication, *HSCT* hematopoietic stem cell transplantation.

^a^*P* values are from Fisher’s exact or Kruskal–Wallis test and compare the two groups.

Of the 12 immunologically responsive AML patients, six were part of the subgroup with highly diverse *TRB* repertoires at diagnosis. In total, 19 out of 51 patients (37.3%) had favorable immune metrics either characterized by a highly diverse pre-AZA *TRB* repertoire and/or by a T cell richness boost after AZA. These patients had significantly longer event-free (*P* = 0.0001) and overall survival (*P* < 0.0001; Supplemental Fig. 9) compared to the rest of the cohort. However, there were no associations between a favorable immunological profile and established clinical characteristics (Supplemental Table 4).

### Patients with favorable BM T cell immune metrics might benefit from a continuation of AZA treatment

AML patients with favorable BM T cell immune metrics either pretreatment (high Shannon diversity index) or day 15 post-AZA (high richness of the *TRB* repertoire) had favorable prognosis when treated with AZA and intensive chemotherapy as well as when only treated with AZA. With the caveat of low patient numbers in the respective subgroups, patients with a favorable BM immunological profile had slightly improved event-free (at 24 months after treatment initiation 66.7% vs 38.5%; Supplemental Table 5) and overall survival (at 24 months after treatment initiation 83.3 vs 60.6%; Supplemental Table 5) when treated with AZA as opposed to AZA and additional chemotherapy. In contrast, in the subgroup without high baseline BM *TRB* diversity and/or richness boost after AZA treatment, the prognosis remained very limited regardless of the applied treatment regimen (Supplemental Table 5).

### T cell TRBV-J gene usage in the AML BM niche after AZA

**Fig. 4:**
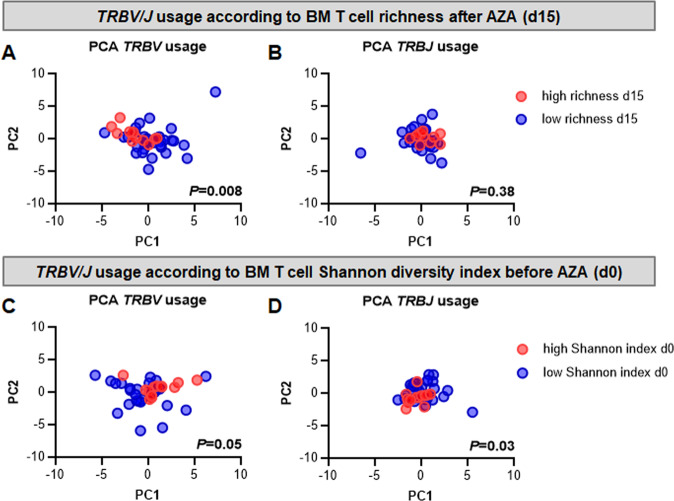
AML patients experiencing a richness boost after azacitidine (AZA) treatment display skewed *TRBV* gene usage. Principal component analyses (PCA) of *TRBV* and *TRBJ* gene usage comparing AML patients with increased T cell repertoire richness after azacitidine to AML patients without immunological response (**A**, **B**) revealed *TRBV* skewing. Similarly, patients with particular diverse T cell repertoires at diagnosis showed differential *TRBV* (**C**) and *TRBJ* gene usage (**D**).

In order to investigate if a specific *TRBV-J* gene usage drift was induced by treatment with HMA, we performed an analysis of the *TRBV-J* gene usages in BM T cells of AML patients before and after the first course of AZA. PCA did not reveal biases in *TRBV* or *TRBJ* gene usage post-AZA in the entire cohort (*TRBV*: *P* = 0.27; *TRBJ*: *P* = 0.64; Supplemental Fig. 10). Yet, when we compared the *TRBV-J* usage after AZA in the patients with the prognostically favorable richness boost upon AZA treatment, we observed significant *TRBV* skewing (*P* = 0.008, Fig. 4A), while *TRBJ* gene usage was not significantly different between both groups (*P* = 0.38, Fig. 4B). Differential *TRBV* gene usage was driven by multiple genes, with *TRBV12-3*, *TRBV5-7*, and *TRBV6-9* being significantly overrepresented in the group of AML patients with a broadened *TRB* repertoire after AZA (Supplemental Fig. 11). AML patients with the most diverse *TRB* repertoires at diagnosis showed significant, but less pronounced *TRBV* and *TRBJ* skewing (*P* = 0.05 and *P* = 0.03, Fig. 4C, D).

### T cell cluster analysis in AML pre- and post-AZA BM and HD BM

**Fig. 5:**
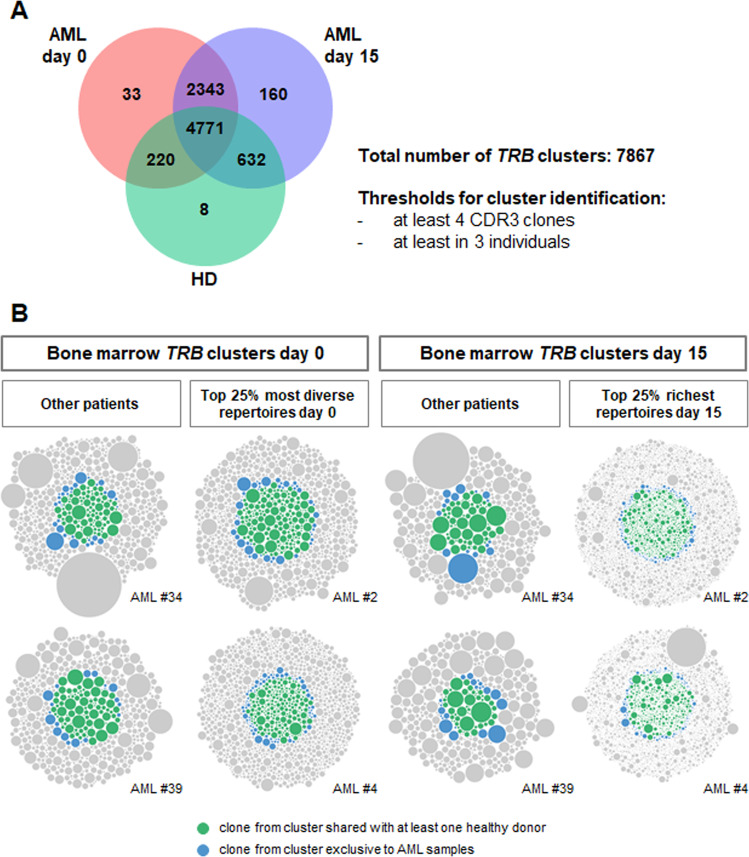
Bone marrow T cell receptor beta (*TRB*) cluster analysis using the GLIPH2 algorithm. Applying the GLIPH2 algorithm we identified *TRB* clusters sharing antigen specificity with relevant overlap between healthy donors (HD) and AML samples (**A**). Thereby, **B** displays exemplary clonal space analysis for four AML patients pre- and post-azacitidine and the contribution of AML specific clusters as well as clusters shared with HD.

To further investigate the AML BM T cell space and deduce potential functionally relevant clonotypes, we applied the GLIPH2 algorithm to our dataset which clusters T cell receptor sequences sharing antigen specificity [39]. To identify *TRB* clusters of pathophysiological relevance for AML, we combined the GLIPH2 analysis with an estimate of the generation probability of each individual V-J recombination using the OLGA algorithm [40]. This analysis identified 7867 *TRB* clusters predicted to share antigen specificity. The majority of clusters (*n* = 4771) were physiological bone marrow T cell clusters shared between all groups of HD and patients (Fig. 5A). A large number of clusters were exclusively found in AML patients (*n* = 2343). Interestingly, there were almost three times the number of clusters exclusively shared between day 15 post-AZA AML samples and HD (*n* = 632) compared to the 220 clusters exclusively shared between AML pretreatment and HD samples suggesting some repertoire normalization upon treatment. AML patients who had particularly rich *TRB* repertoires post-AZA also had a higher absolute number of T cell clusters shared with HD (85.5 vs 11.5 clusters, *P* < 0.0001). Figure 5B displays the clonal space contribution of AML specific clusters and physiological clusters shared with HD for four exemplary AML patients.

**Fig. 6:**
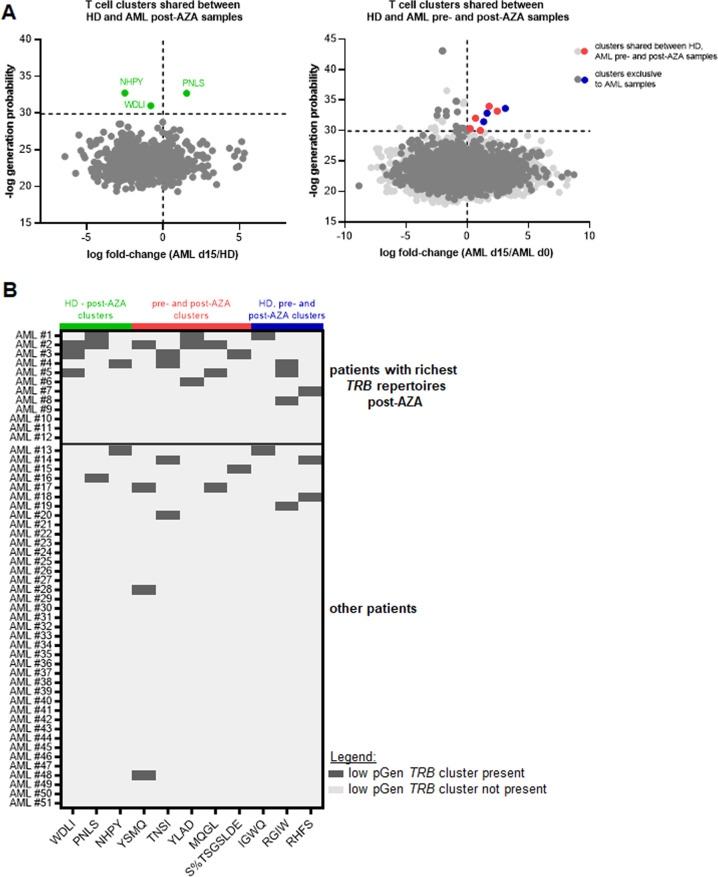
Favorable T cell receptor beta (TRB) clusters with low generation probability (low pGen) are enriched in patients with T cell richness boost after azacitidine. Comparing *TRB* clusters determined with the GLIPH2 algorithm, we identified clusters with low generation probability (−log generation probability >29,89) which were only shared between healthy donors (HD) and AML patients on day 15 after azacitidine (AZA) or enriched in post-AZA samples compared to pretreatment samples (day 0; **A**). These low generation probability clusters were enriched in AML patients with elevated T cell repertoire richness after AZA (**B**).

We also identified several clusters with low generation probability (low pGen clusters, −log generation probability >29,89), which are T cell clusters that are unlikely to emerge by chance but rather result from an immune response to a specific trigger when shared between various individuals [41]. We were especially interested in the low pGen clusters shared only between day 15 post-AZA AML samples and HD (3 clusters) and the low pGen clusters enriched in day 15 post-AZA samples compared to pretreatment samples (Fig. 6A) since these clusters are likely to result from an anti-leukemic T cell response. Interestingly, the patients with highly elevated *TRB* repertoire richness post-AZA showed these low pGen clusters at a significantly higher frequency after the first course of AZA compared to the AML patients without immunological response (1.7 vs 0.4 low pGen clusters per sample; *P* = 0.001, Fig. 6B).

## Discussion

AML remains a fatal disease in elderly patients with dismal long-term survival due to the aggressive phenotype and high treatment-related mortality of the established chemotherapy-based therapeutic regimens [31, 42]. At the same time, AML is an immunologically targetable disease as demonstrated by allogeneic HSCT as a curative treatment option for patients with good performance status [3, 4]. Also, HMA such as AZA, have already been demonstrated to be immunomodulatory [23–28].

The analyzed AML cohort homogenously treated with at least one course of AZA and BM aspirates collected pretreatment and on day 15 of the first AZA cycle provided the unique opportunity to investigate immunological changes in the BM T cell niche in response to HMA. We demonstrate that patients with highly diverse pre-AZA *TRB* repertoires show a favorable prognosis. High pretreatment T cell diversity was also associated with a lower BM blast count (Table 1 and Fig. 2B, *P* = 0.02). However, blast count and Shannon diversity index showed only a very subtle correlation (Supplemental Fig. 12), and the lack of significant prognostic impact of BM blast count in univariate (Supplemental Fig. 5) and multivariate analyses (Supplemental Table 6) indicates that the BM T cell diversity is not a mere surrogate marker of the leukemic burden, but rather represents an important metric of the BM T cell space with its own prognostic significance.

Additionally, we showed that some patients experience a *TRB* richness boost after AZA, which was accompanied by a more diverse and less clonal *TRB* repertoire with a higher absolute number of physiological BM T cell clusters as well as presumably anti-leukemic clusters with low generation probability. Thus, the AZA-induced broadening of the *TRB* repertoire might support antigen-driven anti-leukemic T cell responses, contributing to the favorable prognosis of these patients. The highly diverse repertoire metrics suggest that the BM T cell richness boost very likely results from robust reactivation of thymic T cell output [43].

Patients with immunological response to AZA also had less clonal and more diverse *TRB* repertoires pretreatment, suggesting that repertoires with large and presumably exhausted T cell clones (e.g., CMV T_EMRA_ clones) [44] might not be able to undergo reconstitution of the BM T cell niche in response to AZA. Interestingly, no other clinical characteristics were associated with the broadening of the BM T cell space post-AZA. Of note, a previous study was able to demonstrate *TP53* mutations and mutations in epigenetic regulators (e.g., *DNMT3A* and *TET2*) to be associated with a more exhausted T cell space [16]. However, the limited number of these mutations in our cohort prevented us from further analyzing a potential correlation between the mutational profile and the immunological response to AZA.

In our cohort, patients with favorable immune metrics (high Shannon diversity index pre-AZA and/or high richness post-AZA) experienced longer event-free and overall survival regardless of whether they received only AZA or AZA followed by standard chemotherapy. Consequently, AML patients with very diverse pretreatment *TRB* repertoires as well as patients with “early-on-treatment” immunological response to AZA might benefit from the low-risk profile of AZA treatment compared to intensive chemotherapy while having an equivalent prognosis. Thus, in this subgroup with a favorable immunological profile, AZA treatment could also be the bridging therapy to a potentially curative allogeneic HSCT—which was a possible treatment option in the RAS-AZIC trial—sparing the patients from the additive toxicities of several intensive chemotherapy regimens. In the here analyzed sub-cohort, the percentage of patients receiving an allogeneic HSCT did not significantly differ between the immunological groups and was not significantly impacting on outcome in multivariate analysis, excluding allogeneic HSCT as a confounding factor of the observed favorable outcomes. However, these are retrospective observations in very small patient subsets and, therefore, need to be prospectively validated in future clinical trials. The performance of *TRB* sequencing in a larger AML patient cohort would also help to standardize the technique in this setting which would be a prerequisite for a routinely diagnostic application.

Patients with favorable BM T cell metrics may also represent a subset that could benefit from immunomodulatory therapies in addition to HMA. A phase-II trial in AML patients with relapsed or refractory disease demonstrated an overall response rate of 33% for the combination of AZA with the immune checkpoint inhibitor Nivolumab [45, 46]. Further studies are warranted to investigate T cell metrics as a potential predictive biomarker in this setting.

Taken together, our study suggests that T cell repertoire diversity comparable to healthy BM is associated with a favorable prognosis in elderly patients with AML. Additionally, our study is the first to demonstrate prognostically relevant reshaping of the BM T cell niche and the generation of specific T cell clusters in response to a single treatment cycle with AZA in a subset of patients. Our data thereby imply that these immunological responsive AML patients might benefit from continued AZA treatment as opposed to standard chemotherapy establishing the BM T cell niche as an important prognostic factor as well as a potential predictor of response to different treatment regimens in AML.

## Supplementary information

Supplemental Material

## Data Availability

The reported sequence dataset has been deposited at the European Nucleotide Archive (ID: PRJEB48020).
