# Supplementary material for: Azacitidine-induced reconstitution of the bone marrow T cell repertoire is associated with superior survival in AML patients
Source: Blood Cancer J. 2022 Jan 28;12(1):19. doi: 10.1038/s41408-022-00615-7 (PMC8799690; doi:10.1038/s41408-022-00615-7)
Supplement: Supplementary file 1 — Supplemental Material [file 41408_2022_615_MOESM1_ESM.pdf]

Supplemental Material

"Azacitidine-induced reconstitution of the bone marrow T cell repertoire is associated with superior survival in AML patients"

Grimm et al.

RAS-AZIC OSHO trial investigators

The investigators of the RAS-AZIC trial are: Mathias Hänel[1], Georg Maschmeyer[2], Regina Herbst[1], Song-Yau Wang[3], Christoph Kahl[4], Maxi Wass[5], Herbert G. Sayer[6], and Dietger Niederwieser[3].

Affiliations

[1] Klinikum Chemnitz, Flemmingstrasse 2, 09116 Chemnitz, Germany

[2] Klinikum Ernst von Bergmann, Charlottenstraße 72, 14467 Potsdam, Germany

[3] University Hospital of Leipzig, Johannisallee 32a, 04103 Leipzig, Germany

[4] Klinikum Magdeburg gGmbH, Birkenallee 34, 39130 Magdeburg, Germany

[5] University Hospital of Halle (Saale), Ernst-Grube-Strasse 40, 06120 Halle (Saale), Germany

[6] Helios-Klinikum Erfurt, Nordhäuser Straße 74, 99089 Erfurt, Germany

Supplemental Methods

Definition of Clinical Endpoints

Complete remission (CR) was defined as bone marrow blasts <5%, absence of circulating blasts and blasts with Auer rods, absence of extramedullary disease, absolute neutrophils ≥1.0 x10^9/L; platelet count ≥100 x10^9/L.[1] CRi was defined as all CR criteria except for residual neutropenia (<1.0 x10^9/L) or thrombocytopenia (<100 x10^9/L).[1] Partial response (PR) was defined as decrease of bone marrow blasts to 5% to 25% and a decrease of blast percentage of at least 50% compared to pre-treatment bone marrow.[1] Morphologic leukemia-free state (MLFS) was defined as bone marrow blasts <5%, absence of circulating blasts and blasts with Auer rods, absence of extramedullary disease without the requirement of hematologic recovery.[1] Progressive

disease (PD) was defined as increase in the bone marrow or peripheral blast count or new extramedullary disease. Thereby, the bone marrow blast percentage had to increase > 50% over baseline or the absolute peripheral blood blast count had to increase > 50% to > 25 x10^9/L.[1] Stable disease (SD) was defined as absence of CR, CRi, PR, MLFS, or PD.[1]

Multivariate analyses

Multivariate proportional hazard models were constructed for event-free and overall survival to investigate the prognostic impact of the bone marrow T cell Shannon diversity index pre-treatment (day 0) and the T cell richness after the first course of azacitidine (day 15).

Besides the above mentioned bone marrow T cell immune metrics we also considered the following variables for multivariate analyses: hemoglobin at diagnosis, platelets at diagnosis, white blood count at diagnosis, blast count at diagnosis, blast count on day 15, disease origin (de novo *vs* secondary AML), age at enrollment in the RAS-AZIC trial, presence of a normal karyotype, presence of a complex karyotype, presence of a *NPM1* mutation, presence of a *FLT3* internal tandem duplication, presence of a *TP53* mutation, and performance of an allogeneic HSCT. The variables significant at $\alpha \leq 0.20$ in univariate analyses were included in multivariate analyses. The final models were chosen after forward adjusting for these variables based on the Bayesian information criterion (BIC).

Given the biological importance of the leukemic burden and the potential correlation between bone marrow blast count and T cell diversity we forced the pre-treatment blast count (lowest 25% *vs* the others) into the final models considering the T cell Shannon diversity index as prognosticator.

Allogeneic hematopoietic stem cell transplantation (HSCT) in the RAS-AZIC trial

If a suitable donor was available, allogeneic HSCT could be performed in the RAS-AZIC trial upon discretion of the treating physicians. Azacitidine maintenance could be administered until allogeneic HSCT. In the here analyzed cohort 19 AML patients (37.3%) received allogeneic HSCT. The transplanted patients were also followed up until two years after study enrollment.

Supplemental Tables

Supplemental Table 1. Clinical characteristics of AML patients treated within the RAS-AZIC trial and analyzed in this study.

	**analyzed patient cohort (n=51)**
**age at diagnosis**	
median (range)	69.5 (60.8 – 83.6)
**AML origin, n (%)**	
de novo	28 (54.9)
secondary	23 (45.1)
**BM blasts at day 0, %**	
median (range)	40 (0 – 90)
**BM blasts at day 15, %**	
median (range)	30 (5 – 80)
***NPM1* mutation**	
present	10 (19.6)
absent	41 (80.4)
***FLT3*-ITD mutation**	
present	7 (13.7)
absent	44 (86.3)
**normal karyotype**	
present	23 (45.1)
absent	27 (52.9)
unknown	1 (2.0)
**complex karyotype**	
present	7 (13.7)
absent	43 (84.3)
unknown	1 (2.0)

Abbreviations: AML, acute myeloid leukemia; BM, bone marrow; *NPM1*, *Nucleophosmin 1*; *FLT3*, *fms related receptor tyrosine kinase 3*; ITD, internal tandem duplication; HSCT, hematopoietic stem cell transplantation.

Supplemental Table 2. Clinical characteristics of AML patients treated with azacitidine according to the diversity of their pre-treatment bone marrow T cell repertoire based on the Simpson diversity index.

	AML patients with top 25% most diverse repertoires at day 0 (n=11)	Other AML patients (n=30)	P^a
**age at diagnosis**			
median (range)	68.7 (60.8 – 80.9)	69.4 (61.0 – 83.6)	0.86
**AML origin, n (%)**			
de novo	5 (45.5)	19 (63.3)	0.48
secondary	6 (54.5)	11 (36.7)	
**BM blasts at day 0, %**			
median (range)	14.5 (0 – 70)	30 (0 – 90)	0.12
**BM blasts at day 15, %**			
median (range)	21 (6 – 72)	33 (7 – 72)	0.10
***NPM1* mutation**			
present	2 (18.2)	7 (23.3)	1
absent	9 (81.8)	23 (76.7)	
***FLT3*-ITD mutation**			
present	0 (0.0)	7 (23.3)	0.16
absent	11 (0.0)	23 (76.7)	
**normal karyotype**			
present	4 (36.4)	15 (50.0)	0.31
absent	6 (54.5)	15 (50.0)	
unknown	1 (9.1)	0 (0.0)	
**complex karyotype**			
present	2 (18.2)	4 (13.3)	0.20
absent	8 (72.7)	26 (86.7)	
unknown	1 (9.1)	0 (0.0)	
**allogeneic HSCT**			
performed	6 (54.5)	11 (63.3)	0.48
not performed	5 (45.5)	19 (36.7)	

Abbreviations: AML, acute myeloid leukemia; BM, bone marrow; *NPM1*, *Nucleophosmin 1*; *FLT3*, *fms related receptor tyrosine kinase 3*; ITD, internal tandem duplication; HSCT, hematopoietic stem cell transplantation.

[a]*P*-Values are from Fisher's exact or Kruskal-Wallis test and compare the two groups.

Supplemental Table 3. Multivariate analysis for the here analyzed subgroup of AML patients receiving azacitidine within the RAS-AZIC trial.

Variable	Event-free survival		Overall survival	
	OR[a] (95% CI)	*P*	OR[a] (95% CI)	*P*
**BM T cell richness on day 15** (top 25% *vs* the others)	2.59 (1.02 – 6.56)	0.045	3.39 (0.94 – 12.25)	0.06
**normal karyotype** (present *vs* absent)	–	–	3.12 (1.0 – 9.74)	0.05
**platelets at diagnosis, x10^9/l**	–	–	0.99 (0.98 – 0.997)	0.008

Abbreviations: AML, acute myeloid leukemia; BM, bone marrow.

[a]OR, odds ratio, >1 (<1) indicate higher (lower) chance of survival for the first category listed for the dichotomous variables.

Variables considered for the models were those significant at α≤0.20 in univariate analyses. For event-free survival we included the following variables: BM T cell richness on day 15 (top 25% *vs* the others), performance of an allogeneic hematopoietic stem cell transplantation, and disease origin (de novo vs secondary). For overall survival we included the following variables: BM T cell richness on day 15 (top 25% *vs* the others), performance of an allogeneic hematopoietic stem cell transplantation, presence of a normal karyotype, hemoglobin at diagnosis (g/dl), and platelets at diagnosis (x10^9/l).

Supplemental Table 4. Clinical characteristics of AML patients treated with azacitidine who had favorable immunological profile (high BM T cell Shannon diversity index pre-treatment and/or T cell richness boost after azacitidine) compared to patients lacking these features.

	**AML patients with favorable immune metrics (n=19)**	**Other AML patients (n=20)**	***P*[a]**
**age at diagnosis**			
median (range)	69.2 (60.8 – 80.9)	70.3 (61.0 – 83.6)	0.69
**AML origin, n (%)**			
de novo	12 (63.2)	10 (50.0)	0.52
secondary	7 (36.8)	10 (50.0)	
**BM blasts at day 0, %**			
median (range)	32 (0 – 80)	62 (0 – 90)	0.13
**BM blasts at day 15, %**			
median (range)	27 (6 – 72)	30 (9 – 72)	0.25
***NPM1* mutation**			
present	4 (21.1)	5 (25.0)	1
absent	15 (78.9)	15 (75.0)	
***FLT3*-ITD mutation**			
present	3 (15.8)	4 (20.0)	1
absent	16 (84.2)	16 (80.0)	
**normal karyotype**			
present	9 (47.4)	8 (40.0)	0.63
absent	9 (47.4)	12 (60.0)	
unknown	1 (5.3)	0 (0.0)	
**complex karyotype**			
present	4 (21.1)	2 (10.0)	0.28
absent	14 (73.7)	18 (90.0)	
unknown	1 (5.3)	0 (0.0)	
**allogeneic HSCT**			
performed	10 (52.6)	6 (30.0)	0.20
not performed	9 (47.4)	14 (70.0)	

Abbreviations: AML, acute myeloid leukemia; BM, bone marrow; *NPM1*, *Nucleophosmin 1*; *FLT3*, *fms related receptor tyrosine kinase 3*; ITD, internal tandem duplication; HSCT, hematopoietic stem cell transplantation.

[a]*P*-Values are from Fisher's exact or Kruskal-Wallis test and compare the two groups.

Supplemental Table 5. EFS and OS for AML patients with favorable T cell repertoire immune metrics (pre-treatment high Shannon index and/or high richness after AZA) compared to patients without favorable immunological profile according to the treatment regimen (only AZA vs AZA plus intensive chemotherapy).

	**patients with favorable immune metrics (n=19)**		**patients without favorable immune metrics (n=20)**	
	only AZA (n=6)	AZA + chemotherapy (n=13)	only AZA (n=6)	AZA + chemotherapy (n=14)
**EFS (%)**				
3 months	100	100	80.0	100
6 months	100	84.6	60.0	64.3
12 months	66.7	61.5	20.0	17.9
18 months	66.7	46.2	0	0
24 months	66.7	38.5	0	0
**OS (%)**				
3 months	100	100	80.0	100
6 months	100	92.3	60.0	100
12 months	83.3	84.6	60.0	57.1
18 months	83.3	76.9	0	14.3
24 months	83.3	60.6	0	0

Abbreviations: EFS, event-free survival; OS, overall survival; AML, acute myeloid leukemia; AZA, azacitidine.

Supplemental Table 6. Multivariate analysis for the subgroup of AML patients receiving azacitidine within the RAS-AZIC trial.

Variable	Event-free survival		Overall survival	
	OR[a] (95% CI)	*P*	OR[a] (95% CI)	*P*
**BM T cell Shannon diversity index on day 0** (top 25% *vs* the others)	3.81 (1.37 – 10.6)	0.011	6.97 (1.86 – 26.1)	0.004
**allogeneic HSCT** (performed *vs* not performed)	2.18 (0.99 – 4.83)	0.054	3.53 (1.38 – 9.04)	0.009
**disease origin** (secondary *vs* de novo)	0.50 (0.22 – 1.17)	0.11	–	–
**BM blast count day 0** (lowest 25% *vs* the others)	0.66 (0.26 – 1.69)	0.38	0.39 (0.15 – 1.00)	0.05

Abbreviations: AML, acute myeloid leukemia; BM, bone marrow; HSCT, hematopoietic stem cell transplantation.

[a]OR, odds ratio, >1 (<1) indicate higher (lower) chance of survival for the first category listed for the dichotomous variables.

Variables considered for the models were those significant at $\alpha \leq 0.20$ in univariate analyses and bone marrow blast count on day 0 (lowest 25% *vs* the others). For event-free survival we included the following variables: BM T cell Shannon diversity index on day 0 (top 25% *vs* the others), performance of an allogeneic hematopoietic stem cell transplantation, and disease origin (de novo vs secondary). For overall survival we included the following variables: BM T cell Shannon diversity index on day 0 (top 25% *vs* the others), performance of an allogeneic hematopoietic stem cell transplantation, presence of a normal karyotype, hemoglobin at diagnosis (g/dl), and platelets at diagnosis ($x10^9$/l).

Supplemental Figures

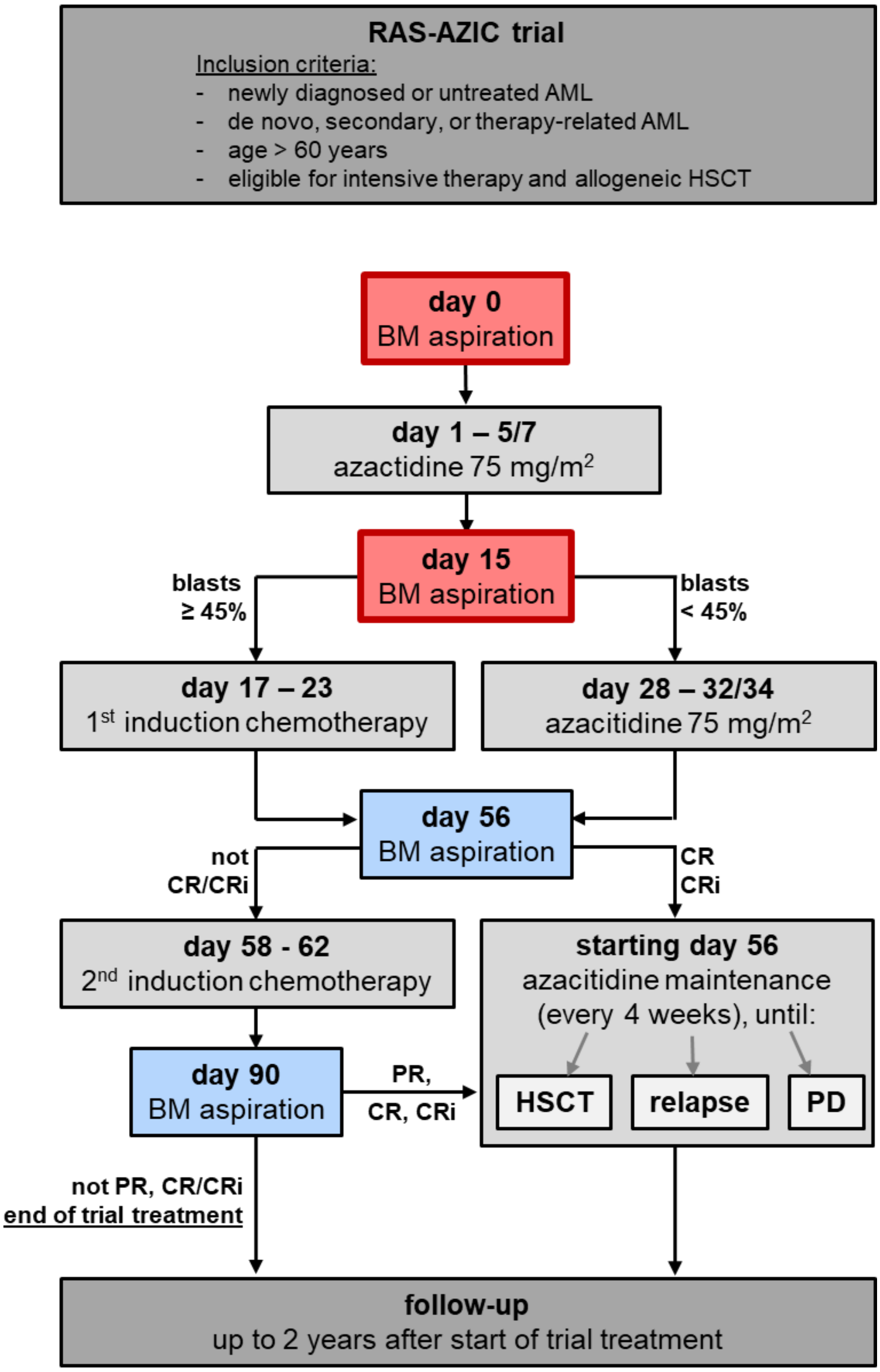

Abbreviations:
AML, acute myeloid leukemia; HSCT, hematopoietic stem cell transplantation; BM, bone marrow; CR, complete remission; CRi, CR with incomplete peripheral recovery; PR, partial remission; PD, progressive disease

Supplemental Figure 1. Flow chart of the RAS-AZIC study design. Samples analyzed in this study were collected on day 0 (before treatment initiation) and on day 15 of the first azacitidine course.

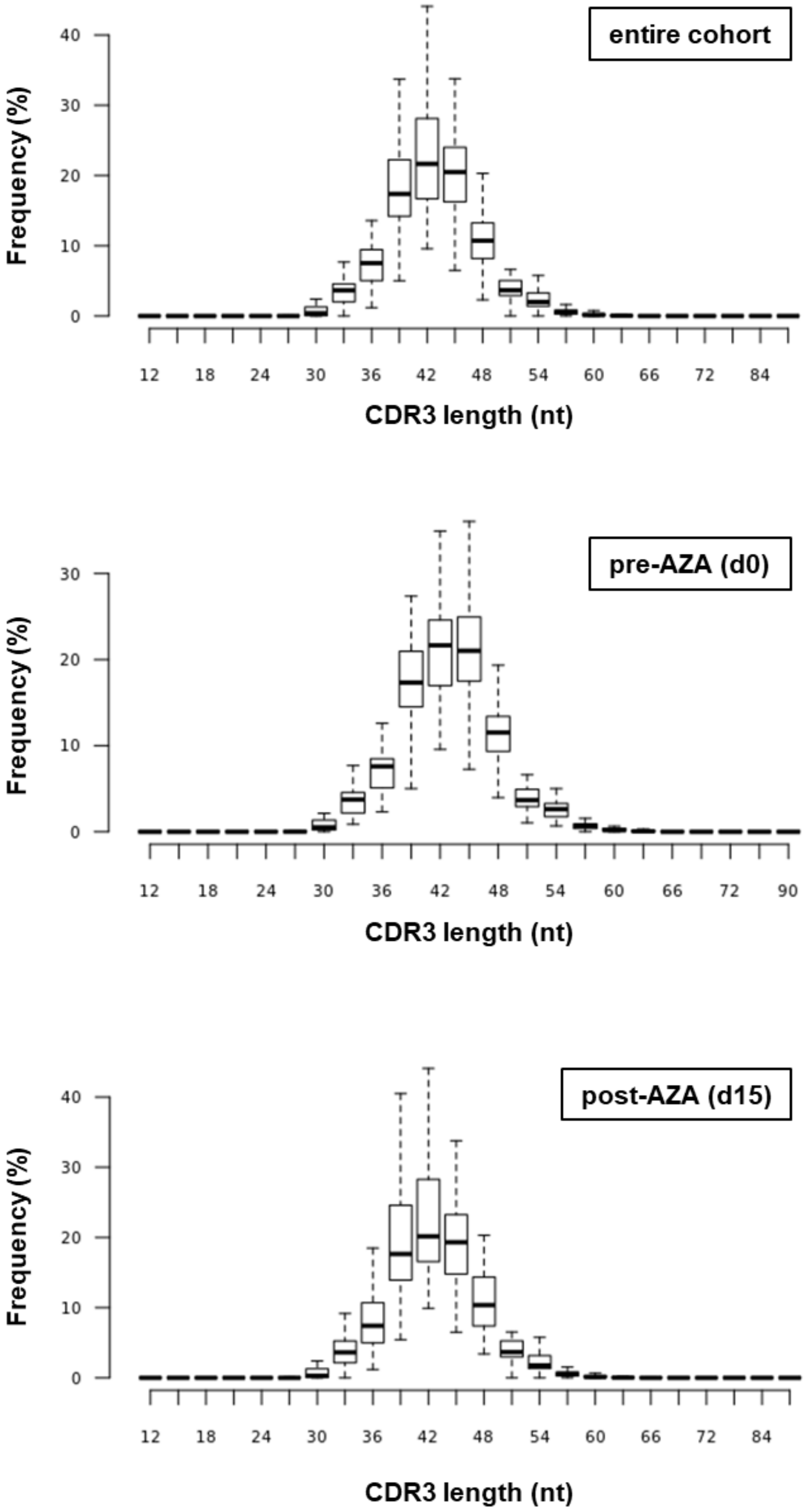

Supplemental Figure 2. Length analysis of the complementarity-determining region 3 (CDR3) displayed Gaussian distribution without significant differences between AML patients pre- and post-azacitidine (AZA).

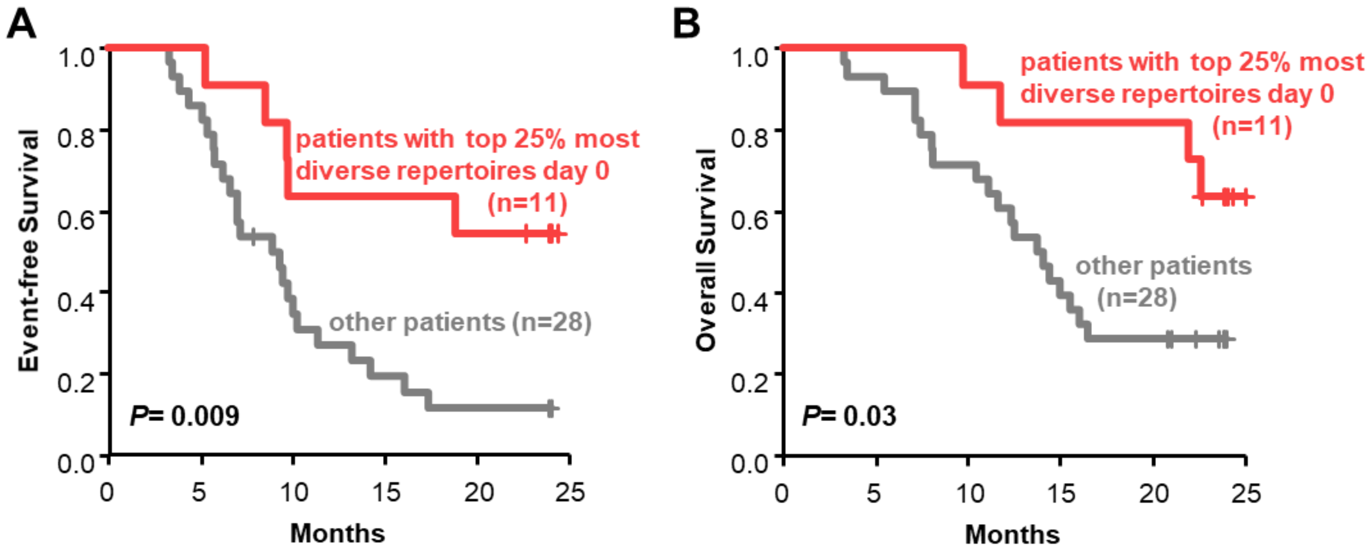

Supplemental Figure 3. After exclusion of AML patients who died within the first 90 days of the RAS-AZIC trial, very diverse bone marrow T cell receptor repertoires before treatment initiation measured by Shannon diversity index (cut-off third quartile of determined Shannon diversity indices) were associated with longer event-free (A) and overall survival (B) when treated with azacitidine and sequential response-adapted intensive chemotherapy.

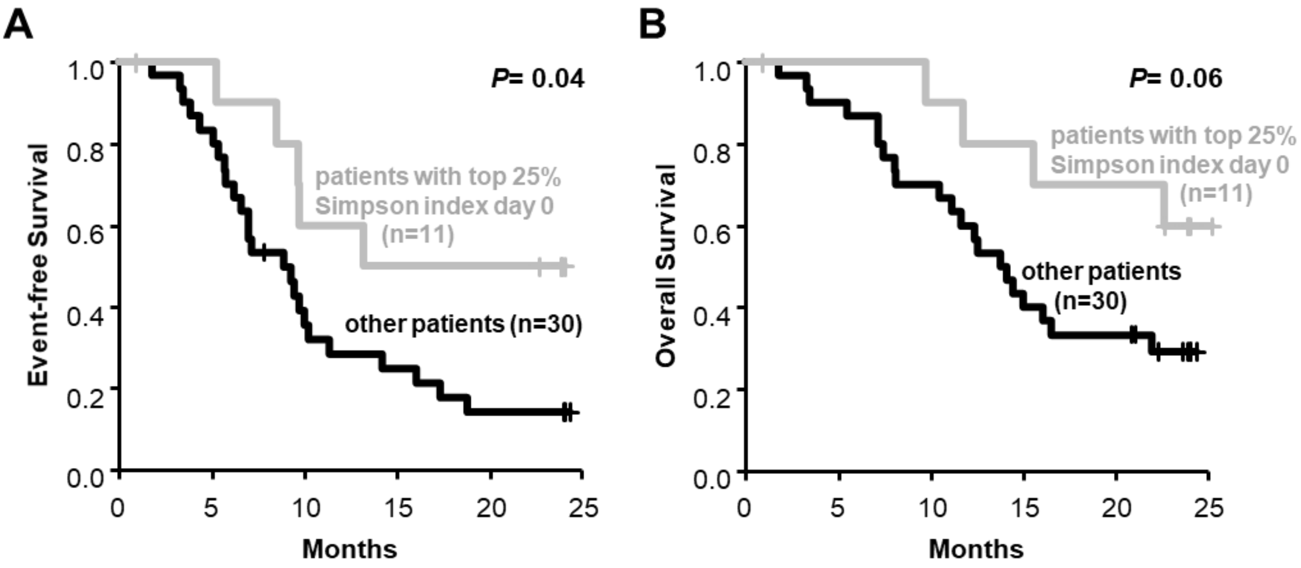

Supplemental Figure 4. AML patients with very diverse bone marrow T cell receptor repertoires before treatment initiation measured by Simpson diversity index (cut-off third quartile of determined Simpson diversity indices) have longer event-free (A) and overall survival (B) when treated with azacitidine and sequential response-adapted intensive chemotherapy.

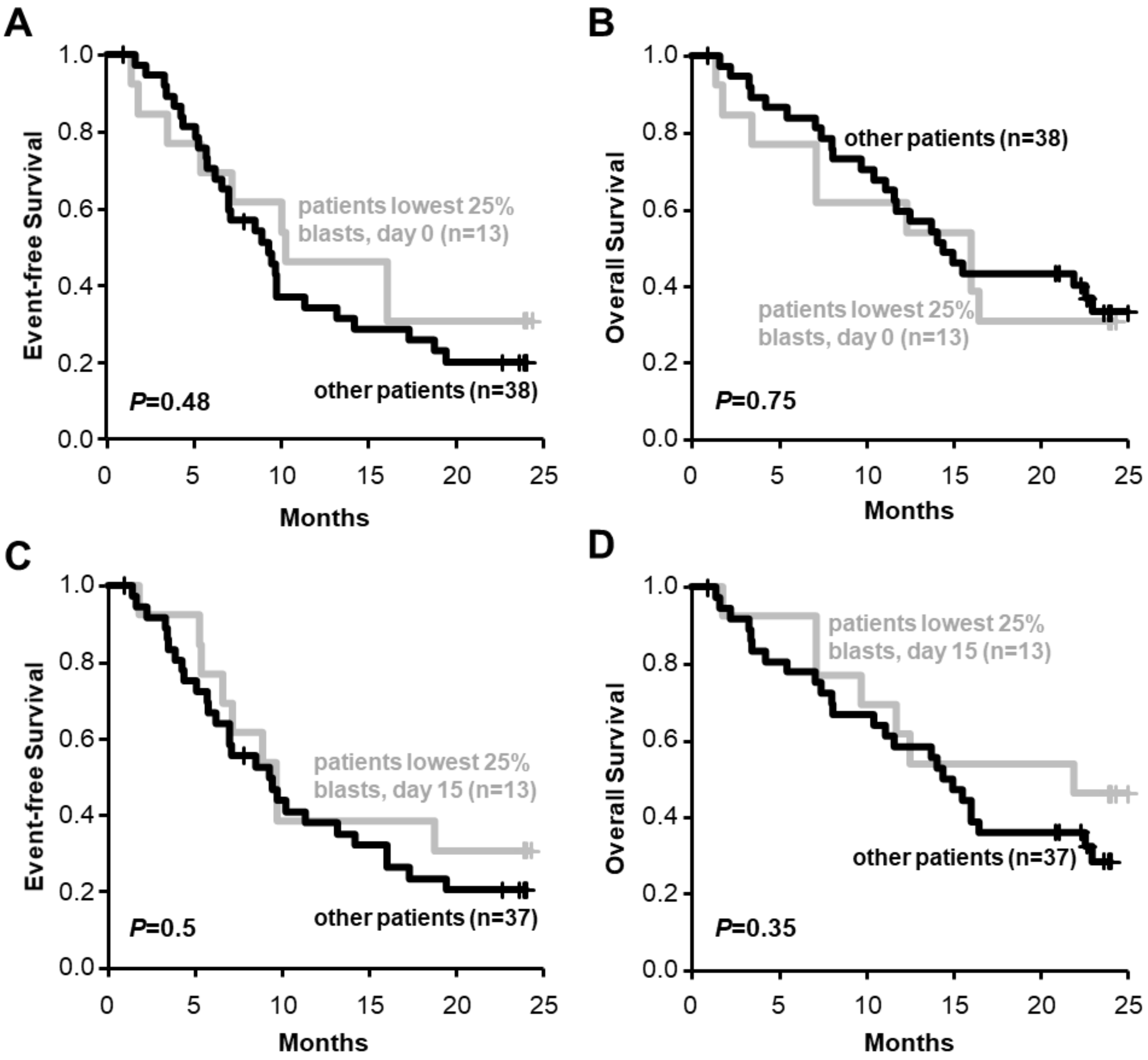

Supplemental Figure 5. AML patients with particularly low bone marrow blast count (cut-off first quartile of the measured blast percentages) before treatment (A and B) or on day 15 of the first course of azacitidine (C and D) do not experience significantly different event-free and overall survival compared to patients with higher leukemic burden when treated with azacitidine and intensive chemotherapy in a sequential and response-dependent approach.

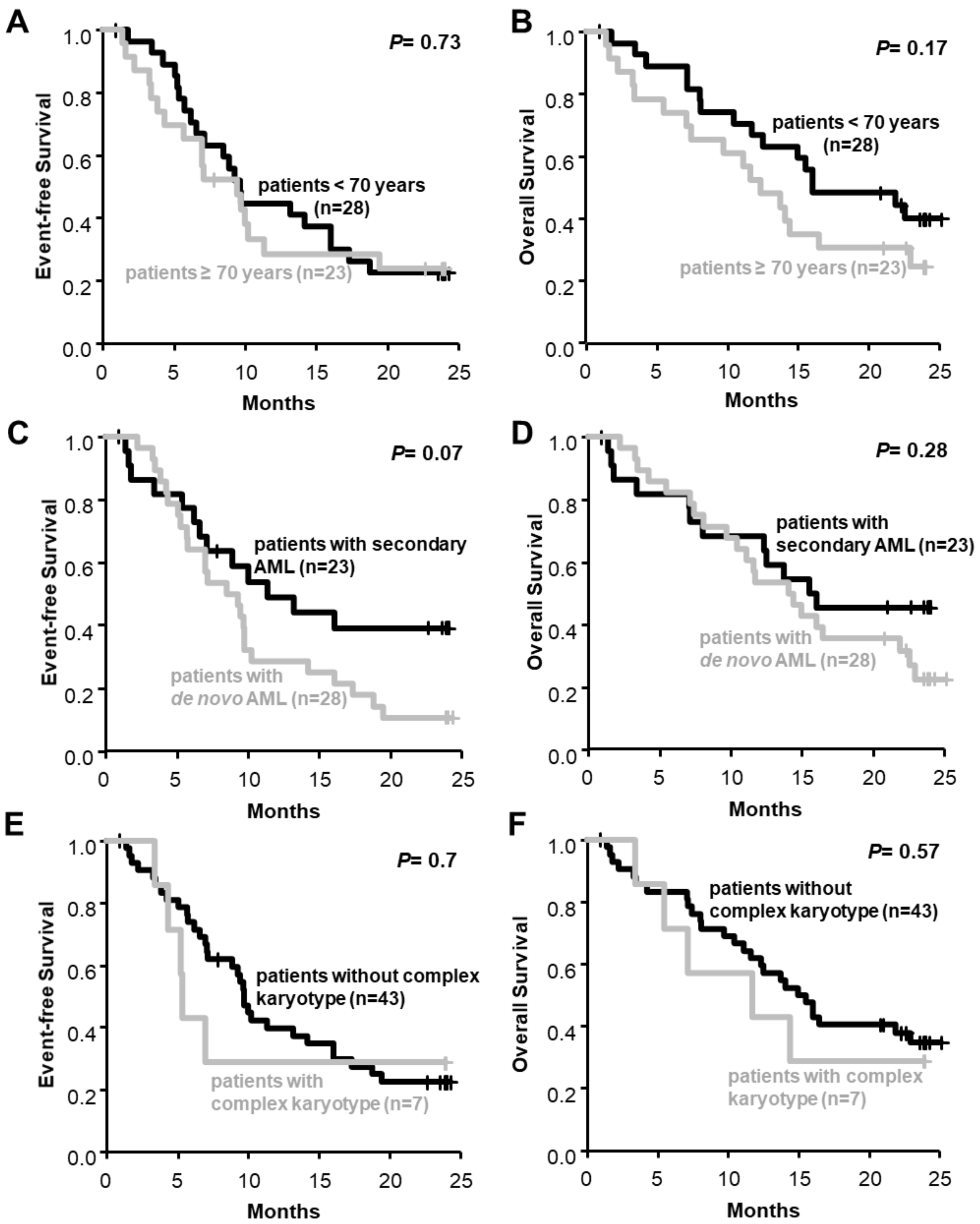

Supplemental Figure 6.

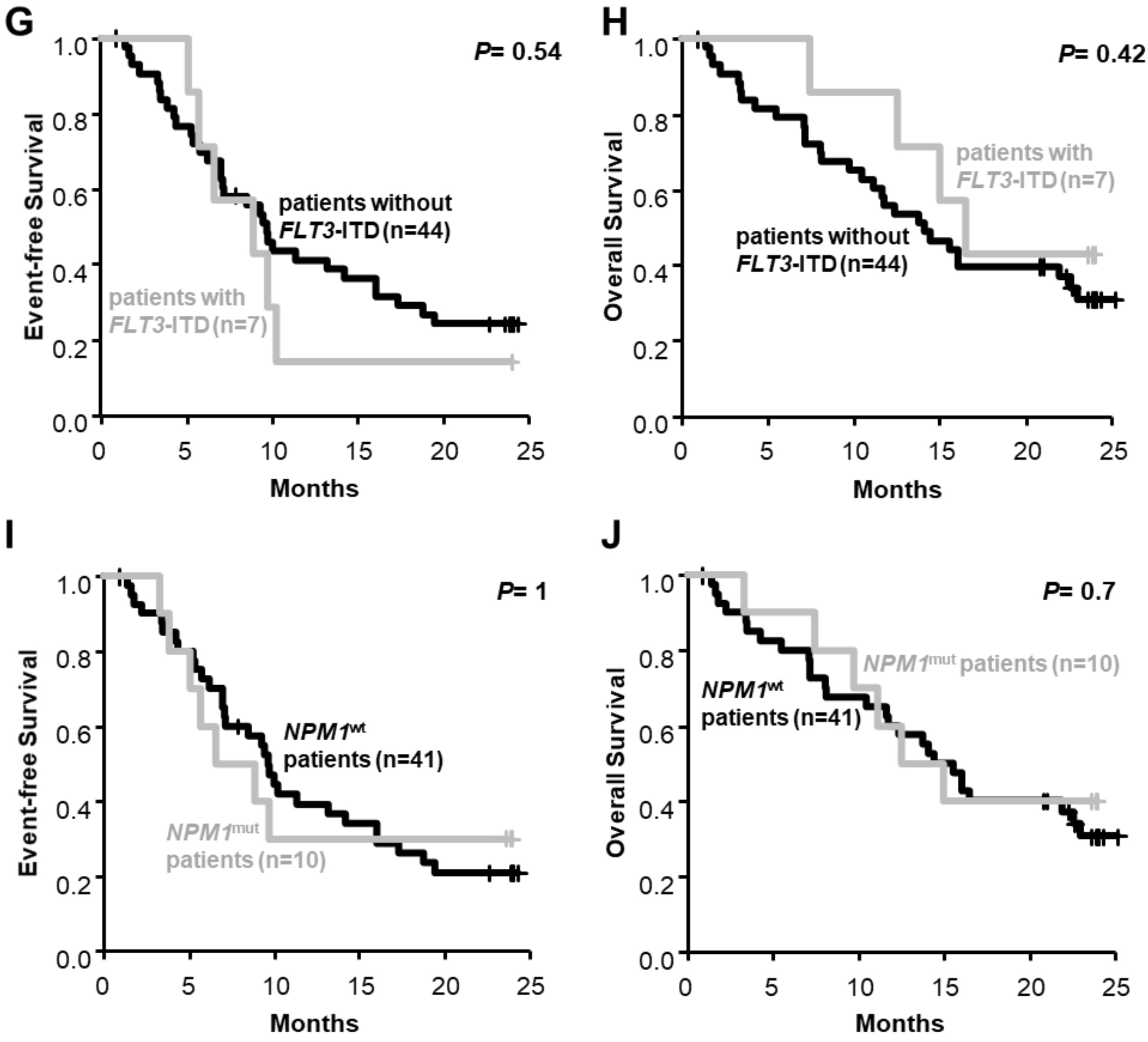

Supplemental Figure 6 (continued). Age at diagnosis (A and B), disease origin (de novo *vs* secondary AML; C and D), presence of a complex karyotype (E and F), presence of a *FLT3* internal tandem duplication (*FLT3*-ITD; G and H), or *NPM1* mutational status (I and J) did not impact on event-free and overall survival in an AML cohort treated with azacitidine and intensive chemotherapy in a sequential and response-dependent approach.

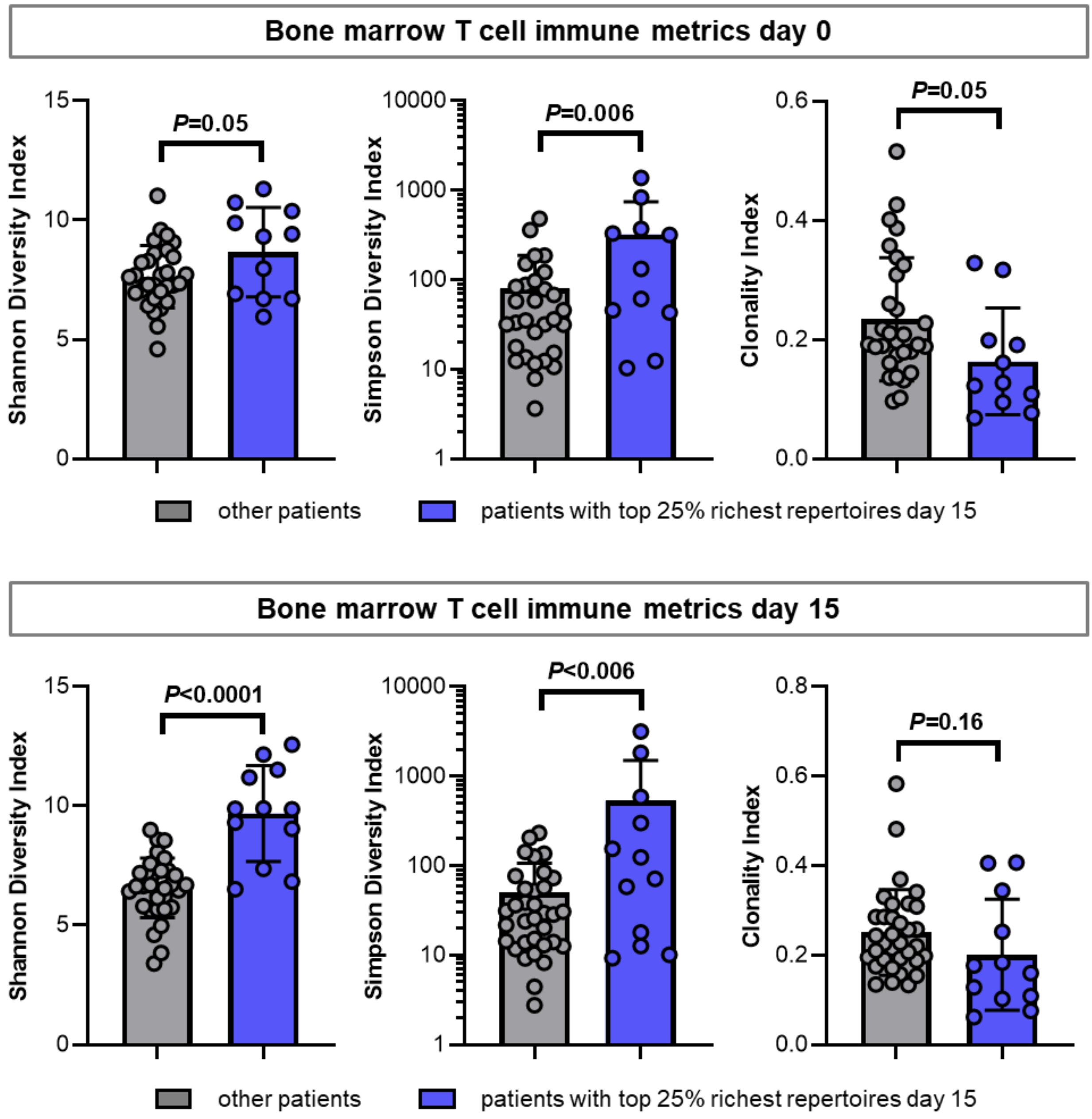

Supplemental Figure 7. Broad T cell repertoire immune metrics of AML patients before treatment and on day 15 of the first course of azacitidine comparing patients experiencing a T cell richness boost after treatment with hypomethylating agents and patients without immunological response.

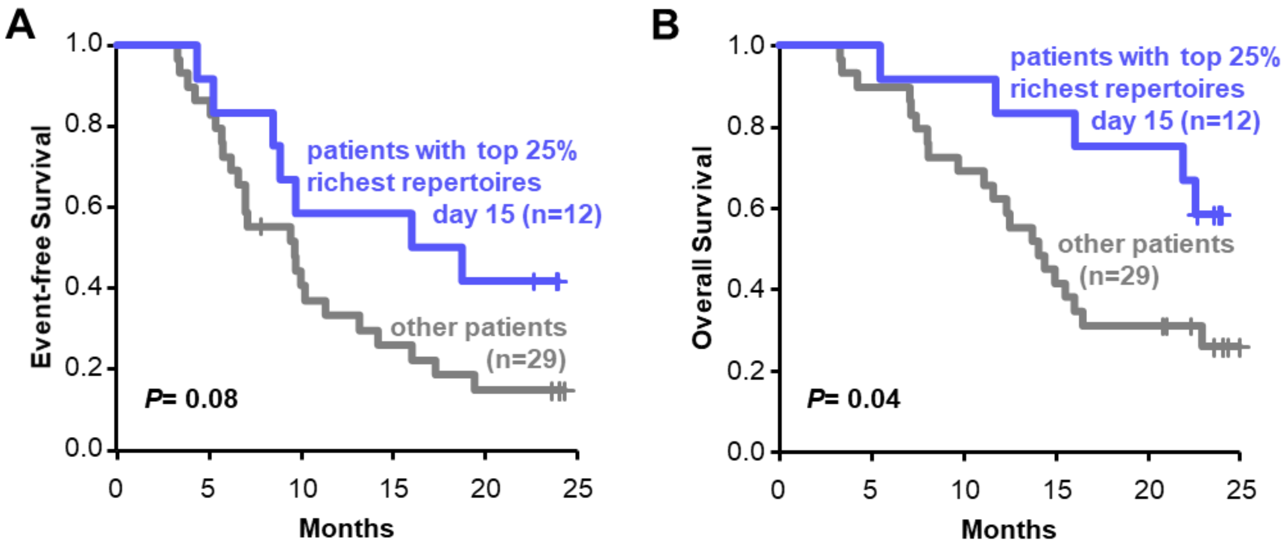

Supplemental Figure 8. Excluding patients who died early (within the first 90 days), a boost of bone marrow T cell richness after the first course of azacitidine was associated with longer event-free (A) and overall survival (B).

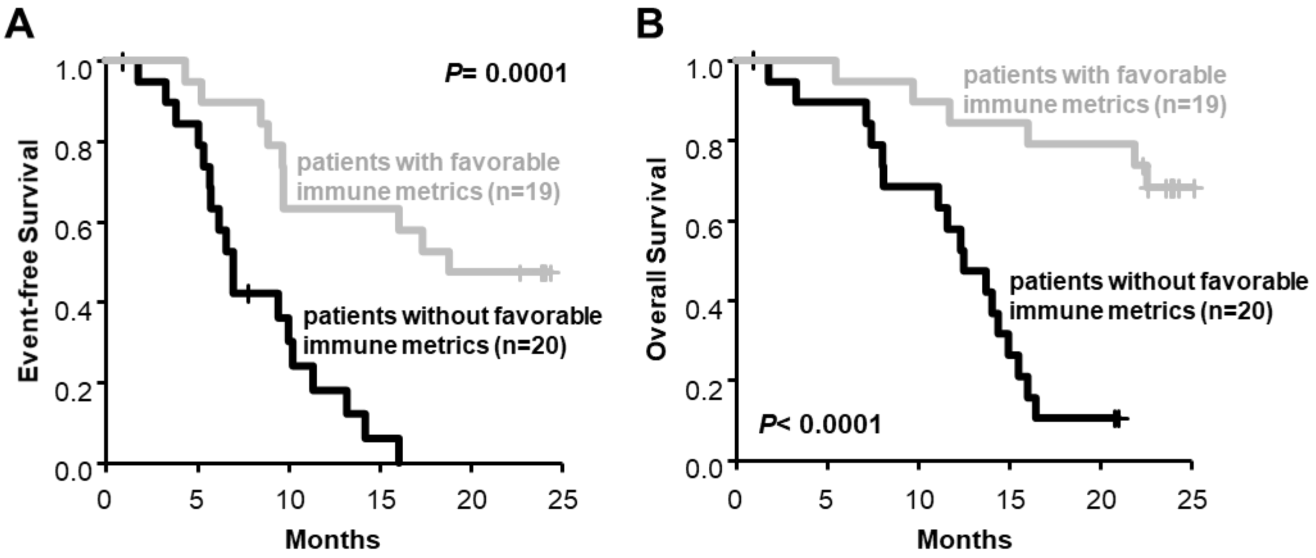

Supplemental Figure 9. AML patients with favorable T cell receptor repertoire immune metrics (high Shannon diversity index pre-treatment and/or azacitidine-induced T cell richness boost on day 15 of the first azacitidine course) had significantly longer event free and overall survival compared to patients without favorable immunological features.

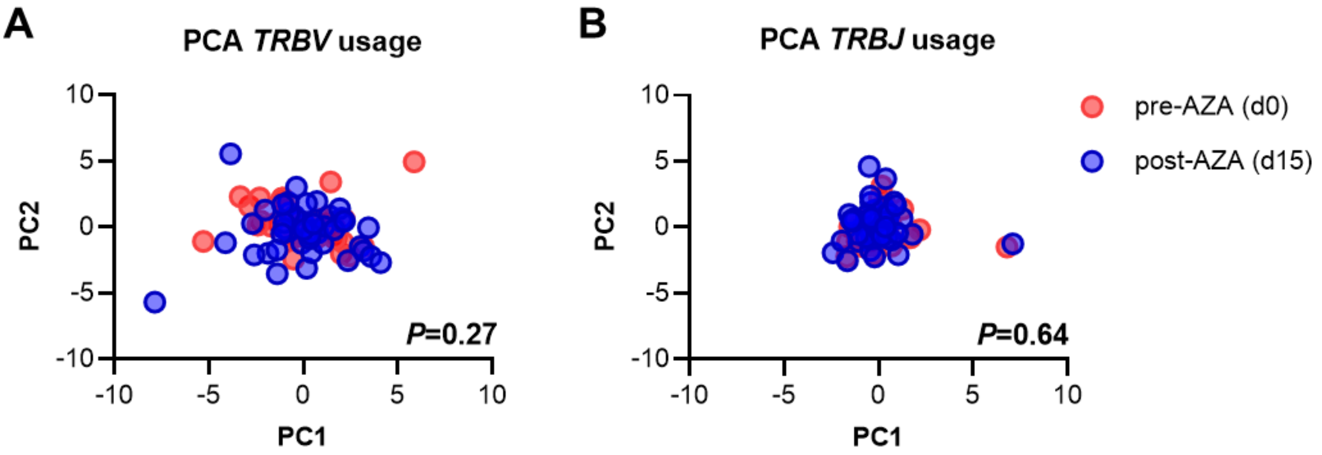

Supplemental Figure 10. Principal component analyses (PCA) of the T cell receptor beta V (*TRBV*) and J (*TRBJ*) gene usage in AML patients before (day 0) and after treatment (day 15) with azacitidine (AZA).

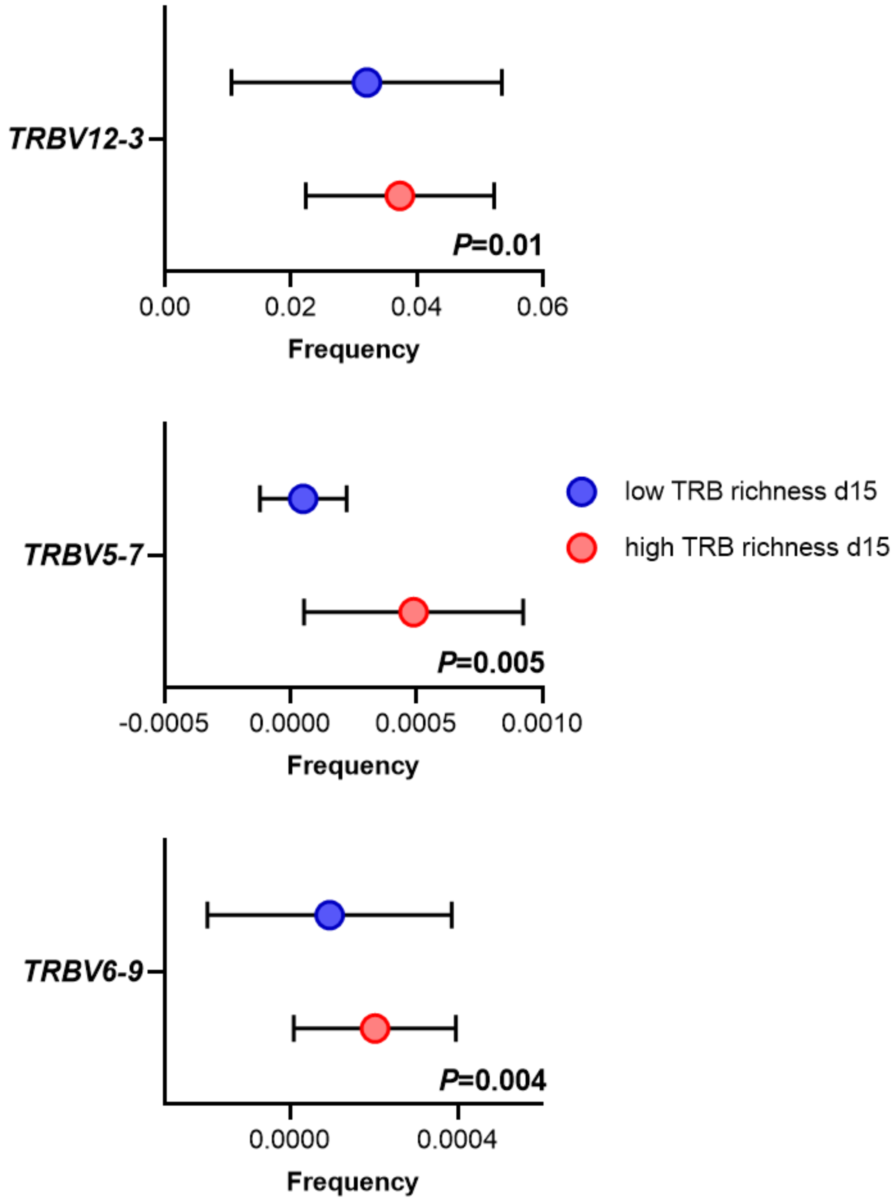

Supplemental Figure 11. T cell receptor beta V (*TRBV*) gene usage was skewed in patients with particularly rich *TRB* repertoires after azacitidine, with the genes *TRBV12-3*, *TRBV5-7*, and *TRBV6-9* being significantly overrepresented compared to patients without *TRB* richness boost on day 15.

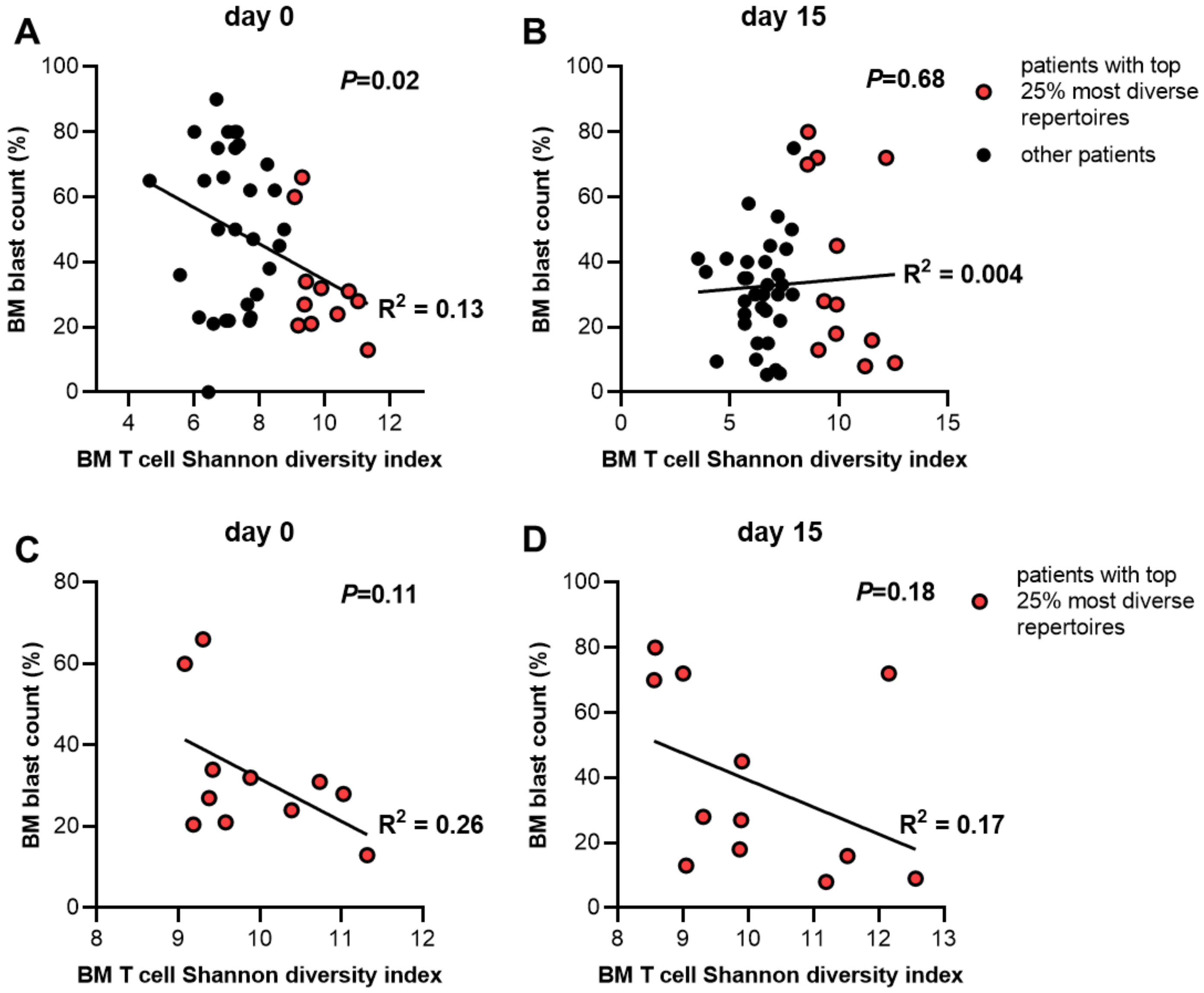

Supplemental Figure 12. Correlation analyses reveal only modest correlation between BM blast count and BM T cell Shannon diversity index on day 0 (A). Analyzing blast count and T cell diversity on day 15 (B) or only in the patient group with the 25% most diverse T cell repertoires (C and D) no correlation is observed.

Supplemental References

1. Döhner H, Estey E, Grimwade D, Amadori S, Appelbaum FR, Büchner T, et al. Diagnosis and management of AML in adults: 2017 ELN recommendations from an international expert panel. *Blood* 2017; **129**: 424–447.
